# Antioxidant Therapy as an Effective Strategy against Noise-Induced Hearing Loss: From Experimental Models to Clinic

**DOI:** 10.3390/life13041035

**Published:** 2023-04-17

**Authors:** Anna Pisani, Fabiola Paciello, Raffaele Montuoro, Rolando Rolesi, Jacopo Galli, Anna Rita Fetoni

**Affiliations:** 1 Department of Otolaryngology Head and Neck Surgery, Università Cattolica del Sacro Cuore, 00168 Rome, Italy; raffaele.montuoro@unicatt.it (R.M.); rolando.rolesi@policlinicogemelli.it (R.R.); jacopo.galli@policlinicogemelli.it (J.G.); 2 Department of Neuroscience, Università Cattolica del Sacro Cuore, 00168 Rome, Italy; fabiola.paciello@unicatt.it; 3 Fondazione Policlinico Universitario A. Gemelli IRCCS, 00168 Rome, Italy; 4 Department of Neuroscience, Reproductive Sciences and Dentistry-Audiology Section, University of Naples Federico II, 80131 Naples, Italy; annarita.fetoni@unina.it

**Keywords:** noise-induced hearing loss, antioxidants, otoprotection, inner ear

## Abstract

Cochlear redox unbalance is the main mechanism of damage involved in the pathogenesis of noise-induced-hearing loss. Indeed, the increased free radical production, in conjunction with a reduced efficacy of the endogenous antioxidant system, plays a key role in cochlear damage induced by noise exposure. For this reason, several studies focused on the possibility to use exogenous antioxidant to prevent or attenuate noise-induce injury. Thus, several antioxidant molecules, alone or in combination with other compounds, have been tested in both experimental and clinical settings. In our findings, we tested the protective effects of several antioxidant enzymes, spanning from organic compounds to natural compounds, such as nutraceuticals of polyphenols. In this review, we summarize and discuss the strengths and weaknesses of antioxidant supplementation focusing on polyphenols, Q-Ter, the soluble form of CoQ10, Vitamin E and N-acetil-cysteine, which showed great otoprotective effects in different animal models of noise induced hearing loss and which has been proposed in clinical trials.

## 1. Introduction

Noise-Induced Hearing Loss (NIHL) is one of the most common hearing disorders, accounting for about 16% of all hearing loss in adult population worldwide [1,2]. It is also one of the most prevalent occupational diseases in the world [3]. For this reason, in recent years, hearing researchers have focused on the possibility of identifying the underlying damaging pathways and to develop effective therapeutic strategies, paving the way to clinical prevention and treatment applications.

Acquired hearing loss can be caused by several environmental risk factors, and one of the most common is exposure to loud noise. NIHL is associated with multiple cochlear damage, including mechanical damage to inner ear structures, oxidative stress with increased free radical production, damage to mitochondria, vascular injury and alteration of ionic concentration in the cochlear fluids [4,5]. Specifically, Reactive Oxygen Species (ROS), as well as Reactive Nitrogen Species (RNS) have been identified as key factors in NIHL pathogenesis, considering that they play a crucial role in triggering apoptotic and necrotic pathways, leading to hair cell death after noise exposure and, consequently, causing sensorineural hearing loss [5,6,7,8]. In this review, based on our findings, we summarize and discuss evaluations of the efficacy of antioxidant molecules, in animal models of NIHL as well as in clinical settings, which showed high protective effects (Table 1). Thus, after a brief overview on oxidative stress damage, we discuss and summarize the effectiveness of antioxidant molecules, such as polyphenol compounds, Q-Ter, Vitamin E, and N-acetil-cysteine that have been also proposed in clinical trials with controversial results suggesting the opportunity of a more targeted approaches against NIHL.

## 2. Cochlear Oxidative Damage in NIHL: A Brief Overview

ROS overproduction is the consequence of the failure of the redox balance between the production of oxidants and the antioxidant defense system activity. Indeed, free radicals can directly oxidize macromolecules, including membrane lipids, enzymes, and nucleic acids, and they are produced in physiological conditions. However, when ROS and RNS production exceeds, this can trigger stress signals, inducing cell death in the principal cochlear structures, such as the stria vascularis [60] and hair cells (HCs) [5,9,52,61,62,63]. Increased mitochondria activity, ischemia/reperfusion, and glutamate excitotoxicity can be considered the potential pathways leading to ROS formation following noise exposure. Specifically, mitochondria are mainly responsible for the ROS formation during their physiological metabolic activity [64]. When the concentration of free radicals increase, ROS are metabolized or scavenged by endogenous antioxidant enzymes, a mechanism including several enzymes (glutathione, glutathione peroxidase and reductase, superoxide dismutase, catalase) that work to reduce oxidative stress and to reestablish redox balance by neutralizing ROS [7].

We know that hair cells in the cochlea are particularly vulnerable to oxidative stress damage because of they have a high metabolic rate, high oxygen consumption, high lipid content, abundant mitochondria, and high energy requirements. Indeed, following noise exposure, mitochondria increase aerobic respiration, using a large amount of oxygen. This leads to a great increase of free radicals, including superoxide (O_2_), singlet oxygen (^1^O_2_) and hydrogen peroxide (H_2_O_2_) [64,65,66,67]. In addition, the exposure to noise can also induce vascular dysfunction, damaging the vascular structure of the cochlea, the stria vascularis. Indeed, noise trauma can cause an ischemia/reperfusion, with a temporary reduction of cochlear blood flow and alterations of in stria vascularis capillaries [68]. In turn, vascular alterations, such as ischemia and reperfusion, can affect also mitochondria metabolism, leading to increased free radical generation and worsening oxidative stress status [68,69,70].

**Figure 1:**
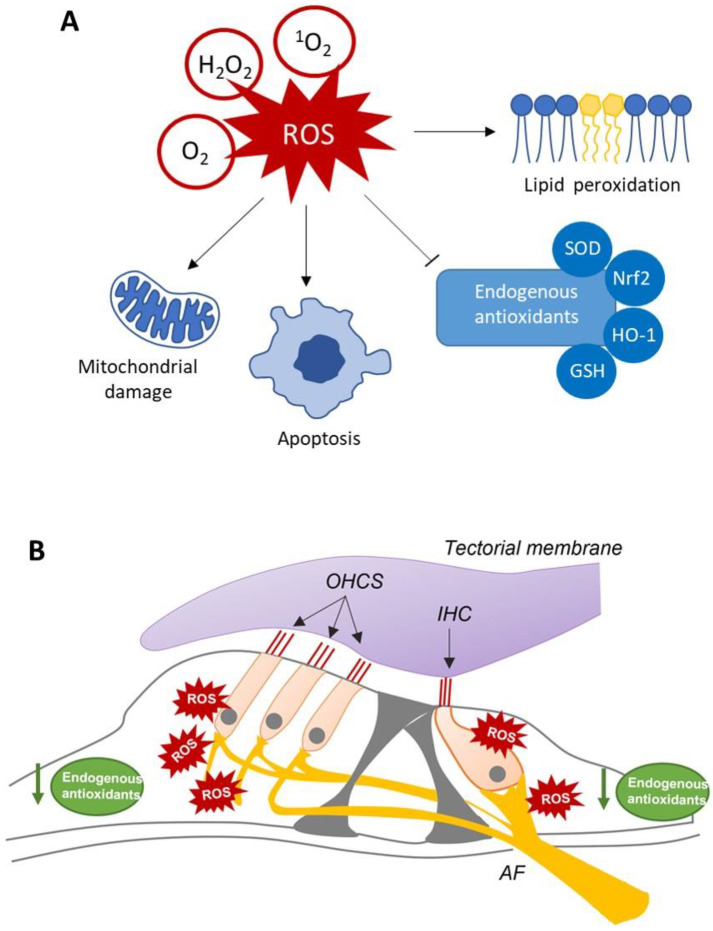
Mechanisms of cochlear oxidative damage in NIHL. (**A**) schematic representation showing the damaging effects of ROS production, including mitochondrial damage, apoptotic pathway activation leading to cell death, lipid peroxidation and reducing endogenous antioxidants defense. O_2_: Superoxide; ^1^O_2_: Singlet oxygen; H_2_O_2_: hydrogen peroxide; SOD: superoxide dismutase; HO-1: heme-oxygenase-1; GSH: glutathione; Nrf2: nuclear factor erythroid 2–related factor 2. (**B**) Schematic representation of the Organ of Corti showing the sensory cells (hair cells), consisting of three rows of outer hair cells (OHCs) and one row of inner hair cell (IHCs), and the neuronal afferent fibers (AF) forming synaptic contact with both OHCs and IHCs. Noise exposure induced a drastic increase of ROS production, leading to redox imbalance and decreasing the endogenous antioxidant defenses.

In physiological conditions, ROS increase is counteracted by an efficient endogenous antioxidant system, so that redox balance in maintained. However, when ROS concentrations increase markedly, cochlear endogenous antioxidant system fail to counteract oxidative stress and become ineffective in scavenging. As a result, cellular damage cannot be repaired, leading to inner (IHC) and, especially, outer hair cell (OHC) death (Figure 1) through the activation of necrosis or apoptosis pathways [5].

Usually, necrosis occurs rapidly and due to lipid peroxidation (a direct consequence of oxidative stress) in the Organ of Corti. In contrast, apoptosis happens slowly, and it occurs several days after noise exposure, due to DNA and protein damage induced by oxidative stress [71].

Several studies have focused on the possibility of leveraging the antioxidant system to counteract oxidative stress and to neutralize the ROS in noise-exposed models. Thus, several studies supported the efficacy of antioxidant therapy to face cochlear oxidative stress and preventing both temporary (TTS) and permanent threshold shift (PTS) induced by noise exposure [38]. Indeed, it has been demonstrated that Vitamins A, C and E in conjunction with magnesium show a great otoprotective activity against hearing loss and cell death [4,72,73]; other researchers reported a successful reduction of NIHL after treatment with several antioxidant molecules, such as Glutathione [74,75], Tempol [76,77]; Ebselen [21,78,79], Alpha-tocopherol [27,28], L-acetylcisteine [29,30,80,81], and D-methionine [82,83]. We have developed in recent years significant experience in evaluating antioxidant and otoprotective efficacy of different exogenous antioxidants, including natural compounds such as polyphenols, against noise-induced damage in animal models. In the following sections, we will highlight the effective protective effects of different antioxidants in NIHL, including phenolic compounds (such as ferulic acid, rosmarinic acid and caffeic acid), Q-Ter, Vitamin E and N-acetylcysteine.

## 3. Effectiveness of Antioxidant Treatments in Animal Models of NIHL

### 3.1. Polyphenols

Polyphenols, especially flavonoids, are chemical components found in vegetables, fruits, grains, and beverage generally derived from fruits, seeds, or herbs, strongly recommend in dietary lifestyle considering that they show several health benefits, including antioxidant and anti-inflammatory properties [84]. Indeed, due to their antioxidant properties, several studies have investigated the protective effects of polyphenol compounds in oxidative stress-related diseases [84,85], including NIHL [86]. Specifically, we studied the protective effects of ferulic acid (FA) in attenuating hearing loss after the exposure to acute acoustic trauma. Our results showed that FA can reduce threshold elevation caused by noise, preventing cell death and apoptosis in the Organ of Corti, by decreasing cochlear oxidative stress and potentiating the endogenous antioxidant system [87]. Indeed, FA otoprotective effect was functionally related not only to its direct ROS scavenger ability, but also to the up-regulation of the cytoprotective enzyme heme oxygenase-1 (HO-1), the microsomal enzyme deputed to heme catabolism [88,89,90]. We also studied the best time schedule of administration to obtain the best FA otoprotective effect against NIHL, and we found that the antioxidant achieved its best cytoprotective capacity if given in the peri-traumatic period, such as before and soon after noise exposure [87]. Other studies supported the otoprotective properties of FA against hearing loss. Indeed, several evidence demonstrated a protective effects of this natural compound in ototoxicity models (cisplatin or aminoglycoside-induced cochlear damage), showing how the ability of this molecule to inhibit apoptosis pathway, reduce mitochondrial ROS formation and potentiate the endogenous antioxidant system is the key molecular mechanisms of its therapeutic efficacy, both in vivo and in vitro models [53,54,91,92,93]. Moreover, among phenolic compounds, we also tested the protective effect of rosmarinic acid (RA) in NIHL [55,62]. RA is commonly found in various *Lamiaceae* plant family, such as rosemary, oregano, thyme or sweet basil [56]. It exerts several beneficial effects, including antioxidant [57] and anti-inflammatory [58] effects. In our study, we found that RA administration in noise-exposed animals was able to activate the redox-sensitive transcription factor nuclear factor erythroid 2-related factor 2 (Nrf2), playing a crucial role in the regulation of cellular defenses against oxidative stress, including HO-1 activation. Indeed, a strong reduction of superoxide and lipid peroxidation, paralleled to increased Nrf2 nuclear translocation leading to an enhancement of endogenous antioxidant enzymes (HO-1, superoxide dismutase and GSH), was observed in cochlear samples of animals exposed to noise and treated with RA [62]. The protective effects of RA on auditory functions were also reported in a model of estrogen deficiency [59], as well as during aging [94] and in ototoxicity [48,95]. Furthermore, we tested another phenolic acid, such as caffeic acid (CA), a phenolic compound able to attenuate and reverse the production of pro-inflammatory factors [49] in addition to the modulation of redox unbalance [50] against cochlear damage induced by noise. Our results demonstrate that CA supplementation was effective in attenuating NIHL and cochlear damage, targeting both inflammatory signaling and cochlear redox balance. Indeed, a strong reduction of inflammatory mediators (i.e., NF-κB and IL-1β), together with reduced oxidative stress and lipid peroxidation markers, and an increased expression of endogenous antioxidant enzymes were found in cochlear samples of animals with NIHL treated with CA [51]. The otoprotective effect of CA is also supported by several studies, showing that this antioxidant can be a valuable candidate to counteract ototoxic damage [96,97,98]. Thus, collectively, these studies supported the use of polyphenols, specifically phenolic acids as valid therapeutic tools to counteract hearing loss and cochlear damage induced by noise exposure. However, one of the main limitations of the use of polyphenols in clinical settings is their low bioavailability, which is largely determined by polyphenol chemical structure and complexity [99] and which is a critical factor for their physiological function [100]. Thus, further strategies are needed to improve polyphenol bioavailability and absorption to obtain significant protective effects in humans.

### 3.2. Coenzyme Q10

Coenzyme Q10 (CoQ10 or Ubiquinone) is a co-factor of the mitochondrial electron transfer chain and is strongly involved in the ATP synthesis in the mitochondria, so that it is high expressed in organs with high metabolism rate [10,11]. CoQ10 is considered one of the most effective antioxidants, preventing lipid peroxidation, increased free radical amount and the consequent damaging modifications of proteins, lipids, and DNA [101]. We previously tested the antioxidant ability of Idebenone, a quinone showing similarities with CoQ10, and we demonstrated that its administration in animals exposed to acoustic trauma significantly reduced cochlear damage induced by noise via counteracting oxidative stress [12,13], in agreement with other studies supporting the strong antioxidant ability of this molecule [14].

We also tested the efficacy of CoQ10 against NIHL and compared the otoprotective effect of CoQ10 with that of the Coenzyme Q10 Terclatrate (Q-Ter), a multi-composite formulation of CoQ10 characterized by a high-water solubility and oral bioavailability. Considering its role in the mitochondrial electron transfer chain, CoQ10 is fundamental in maintaining mitochondrial bioenergetic function, ATP synthesis and redox balance [15]. The reason why we used the CoQ10 water-soluble formulation, Q-Ter, is because, compared to the native form, it shows enhanced bioavailability [16]. Therefore, we evaluated the protective antioxidant effects of Q-Ter in two models of NIHL: acute acoustic trauma (pure tone, centered to 10 kHz, intensity of 120 dB, 1 h) and repeated acoustic trauma (pure tone of 10 kHz, 100 dB, 1 h/day for 10 days).

Our results showed that Q-Ter attenuates NIHL, both in acute and in repeated noise exposures paradigm [52,102,103]. Indeed, Q-Ter was able to reduce oxidative stress markers in the cochlea, such as ROS formation and 4-Hydroxynonenal (4-HNE), a well-known marker of lipid peroxidation [52,102], decrease apoptosis, as demonstrated by TUNEL assay and by the reduced by caspase-3 immunolabelling in the hair cells [102]. Furthermore, we studied the best administration rout, comparing systemic versus trans-tympanic modality, to obtain the best protective effect of Q-Ter in NIHL. Unwanted side effects are the principal limitations that can compromise the effectiveness of otoprotective molecules when administered by a systemic route. Intracochlear or via round window transport administration of the compound may avoid unwanted side effects that may result from systemic application.

Our results confirmed that Q-Ter administration significantly attenuates hearing loss induced by acute noise exposure, and that this functional recovery was paralleled by a significant attenuation of oxidative stress. No significant difference were found by comparing trans-tympanic and systemic treatment, indicating that the effectiveness of Q-Ter antioxidant effect was independent of the administration route [104].

Notably, we also demonstrated that the protective effect of Q-Ter in noise-induced cochlear injury implicated not only oxidative stress, but also inflammation. Several studies reported ROS generation in the cochlea can trigger pro-inflammatory cytokines activation, worsening, in turn, oxidative damage [17,105,106], with a vicious cycle involving the crosstalk between oxidative stress and inflammatory markers [5]. Thus, we tested the ability of Q-Ter in counteracting both oxidative stress and, consequently, inflammatory damage in NIHL. We found that, compared to an anti-inflammatory drug, an antagonist of the IL1 receptor named Anakinra, when blocking oxidative stress with Q-Ter was able to also attenuate not only oxidative stress but also inflammation, leading to decreased expression of NF-κB, an inflammatory transcription factor which is highly sensitive to redox stimuli [103].

Finally, we focused on the cortical consequences of cochlear oxidative damage. Therefore, we tried to understand the effect of peripheral damage induce by noise exposure in cortical auditory regions, such as the auditory cortex. We found that Q-Ter treatment, by reducing hearing loss and cochlear redox imbalance, was able to attenuate morphological damage caused by NIHL in the auditory cortex, by restoring branching architecture and dendritic spine density in pyramidal neurons of layer II/III of the auditory cortex [52]. The overall effective experimental results encouraged the clinical use of this molecule with respect to the other clinically used formulations containing Coenzyme Q 10 because of the potential effectiveness of the terclatration process of this multi-composite molecule. The otoprotective efficacy of CoQ10 has been also documented in experimental models of age-related hearing loss [107] and ototoxicity [104]. As also described in the following section, micronutrients such as vitamins show antioxidant properties, and their efficacy in NIHL has been tested in experimental models [108].

Specifically, Vitamin C administration before noise over-exposure have been demonstrated to be effective in attenuating both TTS [31] and PTS [109,110] induced by noise exposure.

### 3.3. Vitamin E and N-acetylcysteine

Vitamin E and, specifically, its unique form in human bodies, α-tocopherol, is a lipophilic molecule with strong lipid soluble antioxidant properties. Indeed, it can effectively reduce lipid peroxidation, due to its ability to quickly react with peroxyl radicals before they can affect lipid content [22,111,112]. However, the effectiveness of Vitamin E as a scavenger molecule depends on several factors, such as free radical localization and its mobility in the cellular membranes. Indeed, it has been demonstrated that Vitamin E’s antioxidant efficacy decreases as the radical deepens into membranes [39]. Notably, the antioxidant ability of Vitamin E has been confirmed by studies supporting its protective role in cardiovascular diseases [32] and neurodegenerative disorders [7,33,113]. As regards cochlear injury, it has been shown that Vitamin E can counteract oxidative stress-induced damage both in single administration and in conjunctions with other types of antioxidants [12,13,34,35]. Specifically, we previously reported the antioxidant protective effect of Vitamin E in a model of NIHL, caused by acute exposure to noise. Vitamin E was administered in a time window consisting in 1 h before noise exposure and once daily for 3 days. Our results showed that this antioxidant provided a great protection against NIHL, attenuating hearing loss induced by acoustic trauma and decreasing OHC loss. Indeed, ABR recordings were measured at different time points after the acoustic trauma (from 1 h to 21 days after noise exposure), and the functional damage was significantly attenuated in animals treated with Vitamin E with respect to controls [23].

Among all antioxidants, N-acetyl, L-cysteine (NAC) is probably the most studied molecules to counteract cochlear redox imbalance induced by noise exposure. Indeed, the effectiveness of NAC has been evaluated in several experimental conditions, animal models, and dosages [30,36,114]. NAC is able to scavenge H_2_O_2_ and hydrogen radicals and it can positively regulate the maintenance of cellular GSH levels, because of it is a substrate for its synthesis [36]. As for Vitamin E, single or combined treatment with NAC and others antioxidant molecules have been demonstrated to be effective. For example, the combined treatment with NAC and 4-hydroxy phenyl N-tert-butylnitrone (4-OHPBN) has been shown to be effective against NIHL, reducing OHC loss [115]. Interestingly, the protective effect of using NAC in attenuating age-related hearing loss in SAMP8 mice has been demonstrated [116].

Our previous experience suggests that NAC can be effective in attenuating NIHL. Indeed, we examined the protective effects of NAC both as a direct free radical scavenger and as a neuroprotective agent. Its protective efficacy in cochlear injury has been confirmed in several animal models, such as in cisplatin ototoxicity [117] and in NIHL [29,72,81,118]. Animals treated with NAC for three days after acute noise exposure showed a similar temporary loss of auditory function, but a significant improvement in the recovery of CAP thresholds, with respect to noise-exposed no treated mice. The permanent threshold shift, as well as the damage of sensory hair cell, were significantly reduced in the group of animals treated with NAC. The results obtained in our study provide evidence that NAC treatment attenuated threshold shift and hair cell loss caused by continuous noise exposure. Our data are also in agreement with what has been observed in models of ototoxicity [119,120]. The NAC protective effect against cochlear damage can be “direct”, namely related to its ability to inhibit lipid peroxidation and scavenge free radicals, or “indirect” by increasing the intracellular levels of the endogenous antioxidant GSH by acting as a cysteine donor favoring its synthesis. However, in our studies we observed that NAC treatment did not lead to a complete recovery of CAP threshold shifts. This result suggests that in addition to ROS production, other mechanisms also contribute to the noise induced cochlear damage, so that further research is needed, also testing the synergistic protective effects of NAC in combined therapy with other agents. Several factors are fundamental to ensure the antioxidant protection. Among these, the dose, the timing, and the mode of administration of the antioxidant are crucial, so much so that several studies verify the best “therapeutic window” of intervention. Kopke et al. (2000) and Ohinata et al. (2003) demonstrated that NAC can be effective against NIHL alone or in association with other antioxidant if administered before the acoustic trauma [29,37]. Duan et al. (2004) observed protective effects treating animals before and after the acoustic trauma. Our results provide evidence that NAC is efficacious if administered immediately after the noise exposure and in the following days [30]. Thus, the NAC molecule seems to be the most promising antioxidant for the clinical application based on extensive literature of its effectiveness in several neurodegenerative disease models [121]. The efficacy of NAC treatment has been also reported against PTS induced by noise in animal models [29,122,123].

## 4. Clinical Relevance for the Use of Antioxidants

Nowadays, accepted therapeutic approaches against NIHL are lacking. there are no approved medications for patients with NIHL, and the effectiveness of drugs in clinical trials is still controversial. Indeed, notwithstanding the promising results obtained in experimental models, the clinical relevance of antioxidants is still limited because of conflicting outcomes [124,125]. An additional problem to consider is that antioxidants show hermetic effect, with both antioxidant and pro-oxidant properties, based on the dosage used [93,126,127].

This “antioxidant paradox” [126] represents a crucial limitation for clinical translation. Another important limitation is the poor bioavailability of antioxidant molecules, depending on several factors, including genetic polymorphisms [128]. Indeed, not all individuals respond equally to antioxidant therapies, and this may be due in part to genetic variations that affect the way antioxidants are metabolized and utilized in the body [129,130,131].

In the context of antioxidant treatment against hearing loss, pharmacogenetic approaches involve identifying genetic variations that influence an individual’s susceptibility to hearing loss and/or their response to antioxidant treatment. One approach to pharmacogenetic testing for antioxidant treatment of hearing loss involves identifying genetic variations associated with increased susceptibility to hearing loss or reduced response to antioxidant treatment. For example, some studies have suggested that patients with certain variants of genes involved in antioxidant pathways may experience lesser susceptibility to NIHL [132,133]. On the other hand, specific variations in genes involved in antioxidant pathways, such as the superoxide dismutase (SOD) genes or the glutathione peroxidase (GPX) and glutathione S-transferase genes, have been associated with increased risk of hearing loss [18,19,20,133,134,135].Ohlemiller and co-workers demonstrated that the inactivation of *Gpx1* gene increases susceptibility to NIHL and ARHL in mice, indicating an association between genetic impairment of antioxidant defenses and vulnerability of the cochlea [24].Another approach is to identify genetic variations that affect the metabolism or absorption of antioxidant supplements. For example, genetic variations in the genes encoding enzymes involved in the metabolism of vitamin C or vitamin E may influence an individual’s response to these supplements [25,26].Pharmacogenetic approaches to antioxidant treatment of hearing loss are still in their early stages, and more research is needed to fully understand the relationships between genetics, antioxidant treatment, and hearing loss. To overcome these limitations, recent studies have focused on the possibility to develop innovative drug delivery strategies by using drug carriers as nanoparticles, to enhance antioxidant solubility, stability, and absorption [40,136,137], with promising results.

From a translational perspective, we can distinguish the direct antioxidant approach by scavenging ROS from the indirect approach by activating the endogenous defense system in counteracting injury-induced redox unbalance. We described the link between cochlear oxidative stress damage induced by noise exposure and the activation of the Nrf2/HO-1 pathway. Thus, polyphenols such as RA and RA attenuate NIHL, increasing the endogenous antioxidant defenses with the induction/activation of the Nrf2-ARE signaling pathway, in addition to the well-known direct scavenging capability of polyphenols [5]. Targeting Nrf2 may provide a therapeutic option to mitigate oxidative stress-associated diseases including hearing loss. The Nrf2 transcription factor regulates the expression of a myriad of genes involved in the oxidative stress unbalance in several pathologies, therefore it is considered to be a powerful cytoprotective tool for the treatment of different pathologies [138]. Nrf2 activation could be obtained in two different ways, via a direct activation of Nrf2 with natural products, such as polyphenolic compounds, or by using pharmacological Nrf2 inducers. Unfortunately, there are no Nrf2 approved molecules for clinical practice and, regarding ideal Nrf2 natural or pharmacological activators, there are still several concerns in terms of efficacy, bioavailability, safety, and blood-brain/labyrinthine barrier permeability [139].

### Antioxidant Treatment against Noise-Induced Temporary Threshold Shift (TTS)

Experimental findings suggest that the use of antioxidants as a safer alternative to steroids still represents the first-choice treatment for NIHL, given a more promising targeted approach [41]. Thus, according to the theory of redox unbalance in the pathogenesis of NIHL, molecules with antioxidant properties could be a promising effective treatment. However, most human studies have focused on the short-term effects of antioxidants, and additional research are needed to investigate the long-term effect of antioxidants in attenuating PTS in humans. Due to this consideration, studies described thereafter regards specifically the antioxidant effect against noise-induced TTS. Antioxidants that can potentially play a protective role against noise-induced cochlear damage include Q-Ter, NAC and Vitamin E, herein described. The putative use of these molecules is supported by our preclinical studies and, in some cases, by the evidence of protective effects in a clinical setting. However, the therapeutic application of antioxidants in clinical settings is limited by several factors. A major concern is the low bioavailability and absorption, that is, it could be difficult to reach effective antioxidant concentrations in vivo. Indeed, the higher bioavailability and different pharmacokinetic properties of Q-Ter render this molecule a good candidate for clinical treatment. In a pilot study, we monitored the protective effects of Q-Ter in loud sound exposure in volunteer subjects. By evaluating distortion product otoacoustic emission (DPOAEs), a functional analysis allowing to assess hair cell function, we found that amplitude values were significantly increased in subjects treated with Q-Ter before exposure, specifically 1 and 16 h after exposure (white noise of 90 dB HL for 15 min). However, no significant pure tone audiometry (PTA) variations were observed [42]. This data confirmed not only the antioxidant efficacy of Q-Ter in preventing hair cell dysfunction, but also suggest that DPOAEs represent a useful screening test [42].

Moreover, Staffa and collaborators investigated the effect of Q-Ter on recovery time in NIHL and demonstrated that participants who received Q-Ter had a statistically significant improvement in recovery time compared to those who received the placebo. Additionally, Q-Ter was found to be safe and well-tolerated by participants, with no significant adverse effects reported [43]. Interestingly, CoQ10 supplementation has been proposed in an approach of personalized, targeted, and genetically tailored treatment of a mitochondrial cytopathies caused by genetic defects in CoQ10 biosynthesis described in Korean families. Indeed, this condition of CoQ10 deficiency-6 (COQ10D6), related to biallelic COQ6 variants, is characterized not only by kidney failure, but also by sensorineural hearing loss. Interestingly, the CoQ10 supplementation in these patients limited and, in some cases, improved hearing loss although some patients underwent cochlear implantation [44].

The adequate supply of dietary nutrients is essential for the inducers of these enzymes to reach their most effective level. For examples selenium, vitamin C, riboflavin and metals are essential cofactors for antioxidant enzymes, and specifically Vitamin E is considered a major antioxidant in both in vitro and in vivo studies, and gains some support in clinical trials. Indeed, the antioxidant effect of Vitamin E (400 mg twice daily) in the treatment of sudden deafness has been reported, showing a high hearing gain in 75% of patients evaluated [45]. Gopinath and co-workers found that older men supplemented with Vitamin E had a lower risk of developing hearing loss over a five-year period [46]. The authors suggested that the observed association between antioxidant intake and the prevalence of hearing loss may be due to the protective effects of antioxidants against oxidative stress, which is known to play a role to age-related hearing loss. Moreover, a clinical trial conducted by Hatano and co-workers reported the beneficial effect of the combined administration of Vitamin E and Vitamin C in patients with idiopathic sudden sensorineural hearing loss. Indeed, treated subjects showed a reduced the level of reactive oxygen metabolites [47]. Nevertheless, clinical data are often inconsistent, and while several studies support protective effect of vitamins in different clinical conditions, including cardiovascular diseases and cancers, others did not report beneficial effects. In a recent meta-analysis there was no improvement in the thresholds with vitamin E supplementation [140]. It has been also assumed that vitamin E has low toxicity and is not believed to cause serious adverse effects. It has been demonstrated that Vitamin E supplementation of about 4 months with 60–800 IU vitamin E/d had no adverse effects [141]. However, other studies in humans have clearly shown that Vitamin E decreases platelet aggregation and this concern needs to be considered, especially in older patients undergoing antiplatelet and anticoagulant therapies and sometimes an over-prescription of dietary supplementation for oxidative-stress-related conditions, including oxidative Stress-Related Eye Diseases, osteoarthritis and cognitive decline [124,142,143].

Among the antioxidants, NAC is one of the most used and studied molecules, approved by the Food and Drug Administration (FDA) for the treatment of acetaminophen overdose (paracetamol) and as a mucolytic agent in respiratory diseases. It has been recently introduced in some countries, as an over-the-counter nutritional supplement with antioxidant properties and great commercial appeal as a nutraceutical in several conditions, including lung and cardiovascular diseases, psychiatric illnesses, liver and kidney diseases and others [144]. However, few reports suggest its effectiveness in preventing or decreasing NIHL, or showing a protective effect of NAC against permanent hearing loss [140,145,146], despite the fact that the findings are in some cases controversial and that further research are needed. However, in the study by Doosti et al. [147], the authors tested the protective effect of the co-supplementation of NAC and ginseng in workers exposed to loud noise. The results showed that both NAC and ginseng treatments were effective in the amelioration of the auditory threshold. However, the group that received NAC showed significantly greater improvements compared to the ginseng group. In line with these results, the study of Lin et al. (2010) demonstrated that NAC (1200 mg/day for 14 days) orally administered reduced hearing loss induced by noise [148]. A systematic review of literature addressing the effectiveness of vitamin B12, folic acid, and NAC advised some evidence for protective effect on reducing occupational hearing loss; however, the authors carefully suggested that for decisive evidence of vitamin effective therapies, future studies are needed, requiring high precise criteria for noise and for outcome parameters [73]. Finally, several studies showed that NAC, administered alone or in conjunction with steroids, shows a protective effect attenuating sudden hearing loss [149,150,151]. Overall, current data suggest that NAC can be a good antioxidant candidate to counteract acquired hearing loss. Literature findings further validates the hypothesis that NIHL can be treated by using antioxidants. Among the different concerns regarding the use of antioxidants against hearing loss, major issues remain as to how antioxidant defenses work, what factors can limit their effectiveness and how the endogenous antioxidant system can be potentiated through dietary components and pharmaceutical intervention [5].

## 5. Conclusions

**Figure 2:**
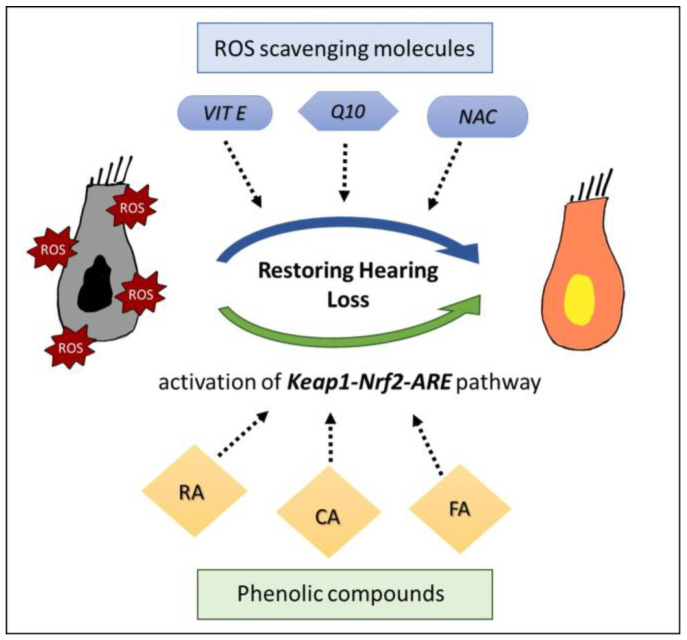
Schematic protective effect of antioxidants compounds against cochlear ROS increased amount induced by noise exposure. Q10 soluble form (Q-Ter), N-acetil-cysteine (NAC) and Vitamin E (VIT E) act directly as ROS scavenging molecules. Phenolic compounds (ferulic acid—FA, rosmarinic acid—RA, caffeic acid—CA) act indirectly by activating the redox-sensitive factor Nrf2. By reestablishing cochlear redox balance, antioxidant protection can counteract molecular mechanisms underlying hair cell dysfunction, attenuating cochlear damage, and restoring hearing loss.

Among all antioxidant compound used in our experience, we focused in this review on several types of molecules: phenolic compounds (ferulic acid, rosmarinic acid and caffeic acid), CoQ10 in its soluble form (Q-Ter), Vitamin E and NAC. These antioxidants have been shown to be effective against NIHL, by counteracting ROS production and attenuating hearing loss (Figure 2). To date, clinical studies have been conducted to evaluate the efficacy of antioxidant molecules in patients with only partial success. This may be due to several factors, including pharmacokinetics and pharmacodynamics differences between animals and humans, as well as the fact that we still have an incomplete understanding of what the effective timing of antioxidant activity is so as to ensure the best protection against cellular and functional damage. This is a challenge to antioxidant clinical application, and further research is needed to find the right intervals to administrate drugs in animals, as well in humans. Further studies will be instrumental in understanding in any depth the pathogenesis and time course of damage, and to evaluate antioxidant drug efficacy, whether alone or in combined therapy with other compounds. This is fundamental in achieving a successful translation from animal research to a clinical setting and to develop new protective strategies against NIHL.

## 6. Future Directions

Considering the higher prevalence of NIHL in the worldwide population and considering that the FDA has not yet approved a drug to counteract hearing loss, to develop effective therapeutic strategies against NIHL is fundamental to improve the quality of life of millions of people suffering from this common condition. Studies reported in this review support the beneficial effects of antioxidant supplementation to prevent, at least partially, this disability. To counteract the detrimental effects of ROS over-production in the noise-exposed-cochlea, it might help to act preemptively. However, it is a matter to be aware of, that pharmacological limitations, such as tolerability, administration route and side effects, must be considered before the approval of its use in this pathology. Even if these, where the use of antioxidants for the prevention of NIHL is a promising area of research, finding the optimal therapeutic agent will remain a challenge for the future.

Future directions may concern the development of novel compounds, combination therapy, targeted delivery systems and personalized medicine approaches. These strategies may improve the efficacy and specificity of antioxidant therapy for NIHL and help reduce the burden of this common hearing disorder.

## Figures and Tables

**Table 1:** Otoprotective effects of antioxidants (↓ decrease; ↑ increase).

Antioxidant Compound	Molecular Mechanisms	Experimental Studies	Clinical Studies
CoQ10, Q-Ter (soluble form) and Idebeneone (Q-Ter analogue)	↓ROS ↓antiapoptosis ↓NF-κB ↓pro-inflammatory cytokines ↑SOD	[9,10,11,12,13,14,15,16,17]	[18,19,20]
**Vitamin E and** α-**tocopherol**	↑GSH ↑SOD	[21,22,23]	[24,25,26]
N-acetyl, L-cysteine (NAC)	↓ROS ↑GSH synthesis ↓NF-κB	[27,28,29,30,31,32,33,34,35,36,37]	[38,39,40,41,42,43,44,45,46,47]
Caffeic acid	↓ROS and RNS ↓NF-κB and IL-1β ↑Nrf2/HO-1 pathway ↑SOD and GSH	[48,49,50,51]	--------
Ferulic acid	↑Nrf2/HO-1 pathway ↓vascular damage	[41,43,44,45,46,47,48,49]	---------
Rosmarinic acid	↑Nrf2/HO-1 pathway ↓NF-κB	[52,53,54,55,56,57,58,59]	----------

## Data Availability

Not applicable.
